# An Examination of the Relationship between Perceived Toxic Leadership and Employee Turnover in the Retail Sector in the UAE

**DOI:** 10.12688/f1000research.171576.1

**Published:** 2025-12-15

**Authors:** Abhishek Shukla, BR Santosh, Sangeetha Vinod

**Affiliations:** 1 School of business, Manipal Academy of Higher Education - Dubai Campus, Academic City, Dubai, United Arab Emirates; 2 Department of Commerce, Manipal Academy of Higher Education, Manipal, Karnataka, India; 3 School of Business, Manipal Academy of Higher Education - Dubai Campus, Academic City, Dubai, United Arab Emirates

**Keywords:** Toxic Leadership, Job Satisfaction, Employee Turnover, Leadership

## Abstract

Toxic leadership or showcasing leadership skills, which are destructive to team spirit and individuals in the workplace, have been noted to significantly influence employee turnover. The purpose of the SLR (systematic literature review) is to investigate the impact of perceived toxic leadership on employee turnover, with the primary focus on the retail industry in the United Arab Emirates (UAE). Fifty journal articles were identified from a systematic analysis of the Science Direct database from 2018 to 2023 and have been included here to investigate toxic leadership, its influence on job satisfaction, organizational commitment, along with strategies that can be adopted to mitigate toxic leadership. This study finds that toxic leadership has a significant impact on turnover intention and organizational commitment of the employees. It affects both the mental and physical well-being of the employees, which leads to poor employee performance. Toxic leadership also negatively impacts the employer brand image and sustainability of the brand. The study outlines that to minimize the negative influence of toxic leadership, companies should provide sufficient consideration for the interests and goals of employees while also fostering emotional intelligence and ensuring that workers feel appreciated and an essential component of the company.

## 1. Introduction

### 1.1 Background for review

Toxic leadership, according to Padmanabhan (2021), is engaging in any leadership style that focuses on leaders not taking into consideration the interests and needs of employees but only on the completion of work objectives or engaging in meeting the interests of leaders or firms. Hence, this aspect involves any attempt to increase workload, job responsibilities, or job time without giving due focus on employees’ social and personal needs. Toxic leadership should be noted to emerge from any part of the organization’s hierarchy and needs to be taken into due consideration by management. This is critical as human resources are a crucial part of any organization. The negative influence of toxic leadership has been established in the study conducted by Felicia et al. (2023). The primary impact of toxic leadership is an increase in stress and a decrease in the physical and mental well-being of employees, which can lead to an increase in the level of burnout and ultimately to the decision to quit the firm to seek better opportunities or the same opportunities that do not cause the same level of stress. Hence, the impact of toxic leadership can be felt across different demographic aspects (age, gender, and work experience); thus, toxic leadership can be felt across all job positions in a firm.

An employee is considered to work more efficiently and continue working with the firm if he or she feels satisfied with the job role and firm, as highlighted in the study by Abd-Ellatif et al. (2021). Toxic leadership might lead to an increase in stress and workload, which negatively influences the level of employee satisfaction and hence makes employees unwilling to continue working. Employees may substitute satisfaction for better compensation, but such compensation is not expected to continue over a longer timeframe. Consequently, job satisfaction has become an inherent attribute that managers must focus upon to ensure employee well-being and target achievement. Another aspect is organizational commitment, which was evaluated by Choudhary & Saini (2021). Organization commitment refers to the psychological state of employees who are committed to the growth of the organization and focused on the achievement of job goals in a dedicated and timely manner. Commitment must be ensured by both management and employees. Management is noted to exhibit a lack of commitment owing to the adoption of toxic-leadership practices, which influence organizational commitment showcased by employees and hence influence their intention to stay with the firm for the foreseeable future. Toxic leadership is a major problem and needs to be addressed through dedicated strategies that, according to Lee et al. (2023a), shall primarily focus on management, focusing on the emotional needs and aspirations of employees and hence striking a balance between organizational goals and employee needs. This can be done either through the introduction of dedicated leadership skills such as transformational and inclusive skills or through the development of emotional intelligence among managers. Therefore, the major focus of each strategy is to ensure that managers can take into account employee needs as well and not cause any physical or mental damage to employees who are an inherent part of the organization.

Employees specifically need moral leadership to maintain a positive workplace culture, enhance psychological wellness, and maintain productivity. According to recent research, many organisations, societies, and nations suffer as a result of toxic leadership, a complex and harmful leadership style marked by unfavourable managerial practices. Further study on toxic leadership is still needed, even with the abundance of literature on the negative aspects of leadership and its effects on workers and organisations Akinyele and Chen (2024).

This research responds directly to the research agenda outlined above by Akinyele and Chen (2024), who suggested that further research in toxic leadership is needed in the context of different countries and work settings, as the authors highlighted that entire organisations, societies and even countries are affected by toxic leadership. By providing insights for leadership development and organisational policy reforms in a crucial and sometimes disregarded industry, this research advances our understanding of how toxic behaviours impact employee well-being, performance, and retention. The study offers novelty by synthesizing fragmented research on toxic leadership in the retail sector of the UAE, a context often overlooked. The SLR was developed primarily using the ScienceDirect database, which has an extensive repository of journal articles, to derive unique inferences from existing studies regarding different aspects of toxic leadership. ScienceDirect database was selected for this study as it provides broad access to excellent, peer-reviewed publications in the social sciences, psychology, and business management. It is the best resource for this comprehensive literature review. Its reliable search capabilities and reputation guarantee thorough coverage of pertinent research. However, one limitation of ScienceDirect is that it mostly contains articles from Elsevier, which may leave out pertinent research from other top databases and publishers.

### 1.2 Review objectives

• To examine the impact of perceived toxic leadership on employee turnover.
• To assess how employee satisfaction and organizational commitment are affected by toxic leadership.
• To investigate how shift timing and organizational structure influence the relationship between toxic leadership and employee turnover. To propose effective strategies to reduce the negative effects of toxic leadership types on employee turnover.

### 1.3 Research questions

• What is the impact of perceived toxic leadership on employee turnover?
• How are employee satisfaction and organizational commitment affected by toxic leadership? How does shift timing and organizational structure influence the relationship between toxic leadership and employee turnover? What are the different effective strategies for reducing the negative effects of toxic leadership types on employee turnover?

## 2. Inclusion and exclusion criteria

### 2.1 Criteria for inclusion

• All journal articles were published during the time period of 2018-2023.
• All the articles were free of full text.
• Each article is in accordance with the objectives of the study.

### 2.2 Criteria for exclusion

• All studies that were published before 2018 and were in different stages of publication were excluded.
• All studies that were not readily accessible or translatable to English were excluded.
• All abstracts, book chapters, conference papers, and entire books were excluded from the study.

## 3. Research methodology

### 3.1 Search strategy

The current research is based on the identification and analysis of relevant journal articles from prominent databases. For the present study, the ScienceDirect database was used, which is based on the arguments of Yasin et al. (2020) and Mourão et al. (2020). ScienceDirect is a prominent database that provides an extensive database of academic research journals and articles in social science and other domains; ScienceDirect databases have high impact scores and hence are considered most relevant regarding the topic being considered for the study. After the selection of the database, different search strings were developed for the manual selection of relevant articles. The following search strings were adopted for the study: toxic leadership and job satisfaction, toxic leadership and employee turnover, job satisfaction and employee turnover, organizational commitment and employee turnover, leadership and employee turnover, and leadership and job satisfaction. Each of the search strings was developed in accordance with the research objectives, and different inclusion and exclusion criteria were subsequently established for the selection of relevant articles for the study. A brief discussion of each criterion and article identified is given below.

### 3.2 Methods for study selection and appraisal

**Figure 1.**
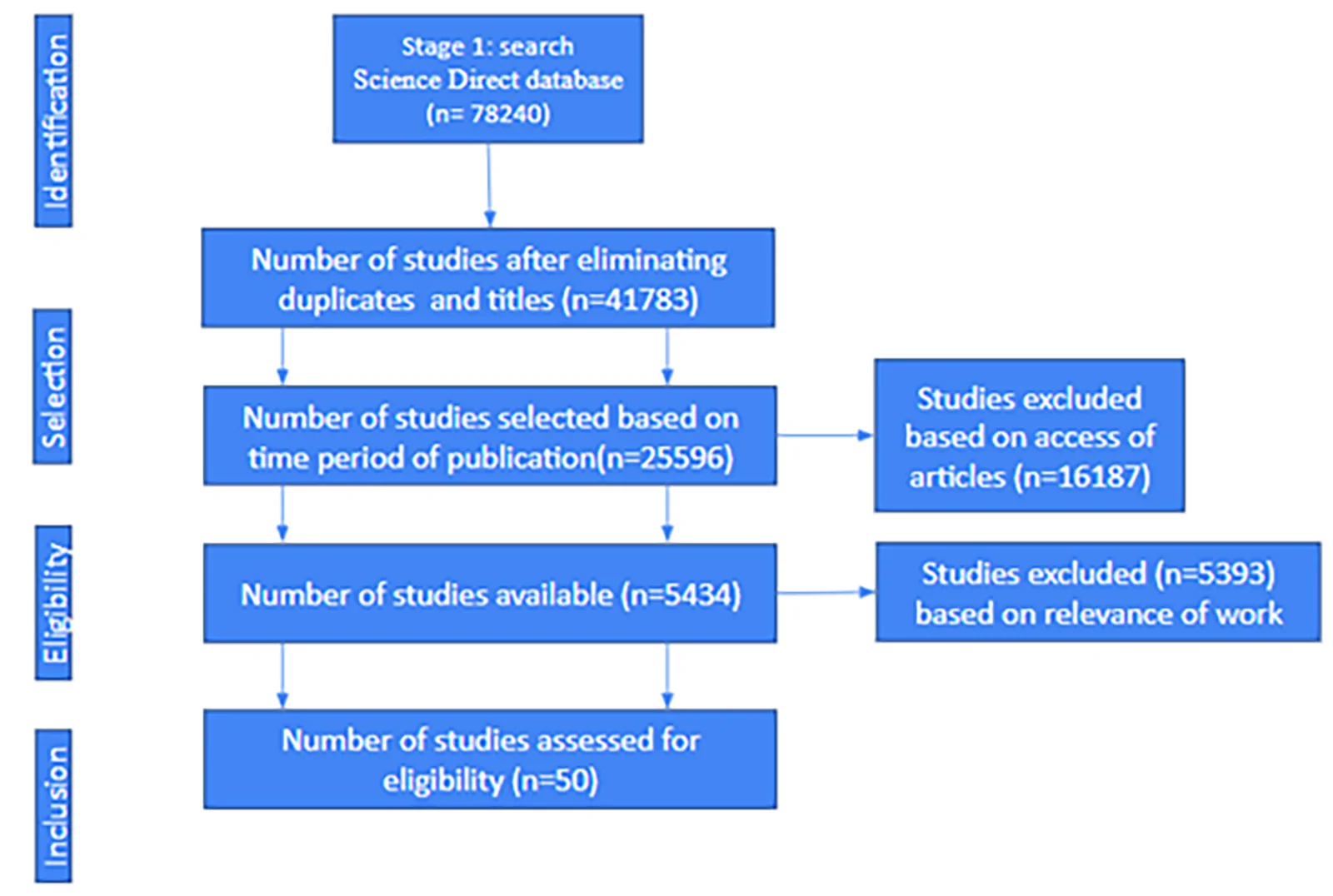
Methodology adopted in SLR: Different steps in performing the research.

The PRISMA research model established in the studies conducted by Page et al. (2021) and How et al. (2022) is the primary research methodology adopted in the study. An initial search of the ScienceDirect database using keyword strings established led to the initial selection of approximately 78240 journal articles. In the subsequent stage, all duplicates and titles were eliminated from the database that was developed in the initial study, which led to the inclusion of only 41783 articles in the database. Next, all the articles selected were reviewed based on the time period of publication (published from 2018 to 2023), which led to the identification of 25596 articles that met the inclusion criteria and were unique. The next step involved the exclusion of all such articles that were not open access (or not accessible by general readers who may not have access to paid journals or databases), which led to the further exclusion of 16187 articles and the selection of 5434 articles for the final database. The abstracts of the selected articles were manually reviewed to ensure that each was in accordance with the research objectives and could provide unique insights, which led to the further exclusion of 5384 articles and the selection of only 50 articles, which are listed in the table below. The PRISMA model below provides a graphical representation of the entire procedure. The process is illustrated in Figure 1.

From the above PRISMA model, we can observe that the initial search using the keywords provides 78240 results. Further out of these studies, 41783 studies were eliminated due to duplication of the research, hence, 25596 studies remained. From these studies, 16187 studies were removed due to a lack of access, resulting in 5434 studies remaining that were publicly accessible. Lastly, 5393 studies were eliminated due to their lack of relevance to the present study, resulting in the selection of the final 50 papers.

## 4. Extraction and synthesis

The extracted data are summarized in **Table 1**. (Extended data)

## 5. Review results and discussion

**Figure 2.**
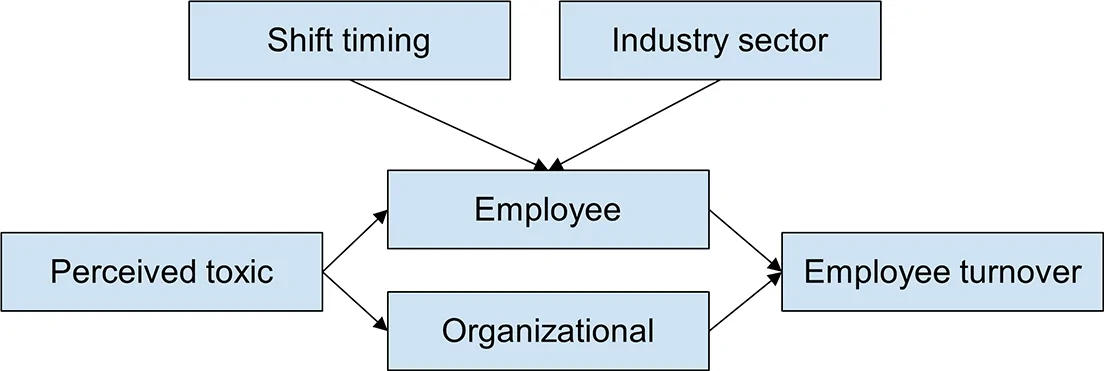
Conceptual framework.

The conceptual framework is presented in Figure 2.

### 5.1 Toxic leadership and employee turnover

In the contemporary corporate landscape, toxic leadership poses a formidable challenge for employees, as underscored by Ali and Ullah (2023). Emphasizing the importance of autonomy and participatory involvement in the workplace, employees find these essential aspects notably absent in toxic leadership environments, thereby fostering an environment conducive to increased employee turnover. Furthermore, Aman et al. (2023) assert that leadership styles significantly impact job-turnover intentions due to their influence on organizational culture. Toxic leadership, characterized by a lack of promotion of a positive organizational culture, contributes to elevated turnover rates across diverse job roles. Cortés-Denia et al. (2023) extend this perspective by highlighting the adverse effects of toxic leadership on employee engagement and commitment to achieving organizational objectives, ultimately propelling individuals to seek alternative employment in organizations that prioritize and respect social aspects of employee well-being.

The detrimental impact of toxic leadership extends beyond turnover rates, as noted by Felicia et al. (2023). Traits associated with toxic leadership contribute to employee burnout and diminish job satisfaction, thereby intensifying turnover intentions across various organizational levels. He et al. (2023) delve into the psychological ramifications of toxic leadership, emphasizing how it negatively influences the psychological attributes and behaviors of employees within the workplace. Considering the work of Holm et al. (2023), toxic leadership, manifested through workplace bullying and indifference to related reports, further impairs the quality of work, ultimately fostering an environment conducive to employee attrition over time.

Hossny et al. (2023) underscore the extensive role of toxic leadership, particularly its authoritarian attributes and self-promotional tendencies, in deteriorating organizational culture and diminishing employees’ willingness to remain in the retail industry. Lee et al. (2023b) contribute to this discourse by highlighting that a myopic focus on meeting job demands without addressing individual job aspects engenders a perception of undervaluation among employees, compelling them to seek alternative roles where their contributions are more recognized and appreciated.

Mehmood et al. (2023b) illuminate the detrimental impact of toxic leadership on employees’ psychological well-being and innovation within the workplace. The study establishes a direct negative correlation between toxic leadership attributes and the innovative output of affected employees. Moreover, toxic leadership qualities contribute to a decrease in employee creativity, while Mossarah (2023) underscores the adverse effects of such leadership on employee compensation and flexibility. The discrepancy between expected and offered salary and flexibility intensifies feelings of undervaluation among employees, fostering an environment conducive to organizational departure.

Obuobisa-Darko and Sokro (2023) identify leaders’ failure to align with employee interests and build mental resilience during challenging circumstances as indicative of toxic and insensitive leadership. This trait correlates with heightened turnover intentions among employees, particularly when confronted with the challenges posed by the pandemic. Padmanabhan (2021) accentuates the association between toxic leadership practices and increased stress levels among average employees, influencing their willingness to remain in the same position within the organization. Rafique et al. (2022) extend this perspective by linking toxic leadership practices, exacerbated by the challenges of the pandemic, to heightened job stress, especially in the context of adapting to hybrid work systems and integrating remote work into organizational processes.

Discussing the impact of toxic leadership on the employees, Salahat and Al-Hamdan (2022) identify a decrease in job satisfaction as a direct consequence of toxic leadership, establishing a correlation between reduced satisfaction and the intention to seek better opportunities elsewhere. Al-Jumaili et al. (2023) shed light on the pervasive issue of excessive workload as a common variable associated with toxic leadership. It becomes evident that toxic leadership practices encompass stress, reduced job satisfaction, and an increased workload, collectively impacting the workforce ( Goetz and Wald (2022); Jena et al. (2018)).

While considering the attributes of toxic leadership, Noor et al. (2023) identify gender discrimination and favouritism as prominent toxic leadership attributes that contribute to turnover among specific employee segments that perceive inadequate opportunities for advancement. On the other hand, Sokal et al. (2021) emphasize the lack of support from top management, whether in the absence of a developed strategy or failure to consider middle management proposals, as a toxic leadership practice. Discussing the personality traits of the leaders, Brender-Ilan and Sheaffer (2019) highlight the narcissistic traits exhibited by leaders as another facet of toxic leadership, emphasizing the imperative for prompt and effective management of such traits across all hierarchical positions within the organization.

### 5.2 Influence of employee satisfaction and organizational commitment on the impact of toxic leadership on employee turnover

Abd-Ellatif et al. (2021) assert that employee satisfaction has been adversely affected by heightened economic apprehensions since the COVID-19 pandemic. Within this context, toxic leadership exacerbates concerns among employees, amplifying fears about potential job impacts. Additionally, Adiguzel et al. (2020) posit a close correlation between the quality of leadership exhibited by managers and employee satisfaction. The failure of managers to instil motivation, coupled with detrimental behaviors, directly influences job satisfaction. Bezdrob and Šunje (2021) highlight the transient nature of job satisfaction, emphasizing the dynamic influence of leadership styles that necessitate ongoing monitoring.

Choudhary and Saini (2021) establish the pivotal role of organizational commitment in shaping job satisfaction. Recognizing the profound influence of organizational commitment on both employee turnover and job satisfaction due to toxic leadership underscores the necessity of considering organizational commitment when evaluating leadership impacts. Folakemi et al. (2018) extend this perspective by elucidating the influence of transformational and transactional leadership styles on both organizational commitment and job satisfaction, revealing universal applicability across permanent and temporary organizations and diverse industries.

Inegbedion et al. (2020) discern the differential impacts of toxic leadership attributes on job satisfaction, with certain attributes exerting a more pronounced influence, such as increased workloads and inadequate staffing levels, leading to diminished employee satisfaction. Kaymakcı et al. (2022) correlate the positive impacts of all variables on organizational performance, innovation, and a culture of innovation, emphasizing the necessity of mitigating toxic leadership attributes for organizational goal achievement. Kim et al. (2023) accentuate the universal nature of such influences across various pay levels and job positions, underscoring the pervasive impact experienced by all employees.

Labrague et al. (2018) contribute valuable insights into the universality of these influences across demographic variables, including gender, education, and work experience, reinforcing the generalizability of the relationship. Li et al. (2020) posit job satisfaction as a significant mediating variable in the nexus between various leadership styles and the intention to leave a job, further emphasizing its centrality in leadership impact assessments. Ly (2023) underscores the positive influence of inclusive leadership attributes on organizational commitment, suggesting the imperative for management to cultivate such attributes. Ryu et al. (2020) elucidate the emotional connection between employees and the organization, positing that negative satisfaction and commitment aspects significantly increase the likelihood of employee departure. In contrast, Van Rossenberg et al. (2022) challenge conventional notions of commitment as static, arguing for a dynamic understanding, while Jena et al. (2018) emphasize the interplay between employee satisfaction, commitment, and trust in management actions as key factors in reducing employee intentions to seek better opportunities elsewhere.

### 5.3 Toxic leadership mitigation strategies

Ayça (2019) advocates a substantive approach to ameliorate the ramifications of toxic leadership within the retail industry by intensifying efforts to enhance organizational job satisfaction. Embracing leadership styles, such as authentic leadership, proves instrumental in mitigating the adverse effects stemming from toxic leadership attributes. On the contrary, Brender-Ilan and Sheaffer (2019) propose a strategy in which, without necessitating a radical shift in leadership style, managers are encouraged to actively cultivate autonomy among subordinates in various job functions. This proactive measure serves to attenuate negative subordinate reactions resulting from toxic leadership.

Eliyana and Ma’arif (2019) endorse the adoption of diverse transformational leadership styles, citing their substantial impact on both job satisfaction and organizational commitment. This multifaceted influence, in turn, positively affects employee performance. Hirschi and Spurk (2021) posit a strategy focused on employee advancement and career progression, acknowledging potential drawbacks such as employee migration for superior opportunities. Simultaneously, prioritizing psychological well-being and demonstrating elevated trust in employees, as suggested by Jena et al. (2018), represent pivotal strategies for fostering employee engagement and a sense of organizational value. They also suggested that the HR department must avoid nepotism of any type while practising openness. Improving corporate culture and implementing forward-thinking HR procedures can promote pleasant working relationships between management and staff.

Khizar et al. (2023) underscore the significance of ethical principles and leadership adoption, emphasizing their critical role in guiding managerial actions. These principles align with organizational objectives while addressing the interests of various stakeholders, including employees. Complementing ethical considerations, Lee et al. (2023b) stress the importance of leaders developing emotional intelligence to comprehend employees’ social and psychological needs, collectively contributing to the mitigation of toxic leadership attributes. Lee et al. (2021) underscored social support, particularly in critical situations such as a civic pandemic, as essential for alleviating fear among employees and fostering heightened customer loyalty.

Mittal et al. (2022) highlighted the cultivation of employee brand love as a strategic imperative that positively impacts employee engagement and commitment while diminishing the propensity of employees to depart due to adverse leadership attributes. Creating a friendly workplace environment and affording flexibility, as advocated by Olubiyi et al. (2019), serves as a pivotal strategy applicable across all job positions, precluding perceptions of discrimination and favouritism. Salau et al. (2018) propose the adoption of transformational leadership attributes as a means to instil reassurance among employees impacted by toxic leadership, thereby redirecting their focus toward organizational goals. In order to create an environment of comfort, managers and supervisors must link the vision to a plan for achieving it and work with the staff to create an engaging and challenging vision.

Slatten et al. (2021) posit a significant strategic avenue wherein employees aligning their personal values with company values may establish a deeper connection with the organization. Such alignment potentially fosters increased engagement in management activities. This is because employees actively seek to diminish the adverse effects of prior toxic leadership practices. Furthermore, Wei et al. (2023) assert that the implementation of career advancement and training programs, including formal upskilling initiatives from dedicated certificate-providing institutions, is an effective measure for mitigating negative repercussions. Notably, these interventions directly benefit employees occupying lower and middle positions within the organizational hierarchy.

Coronado-Maldonado and Benítez-Márquez (2023) advocated the cultivation of emotional competence and emotional intelligence among leaders as a favourable strategy. This development equips leaders to navigate complex situations, ranging from the challenges posed by a pandemic to geopolitical conflicts such as the Ukraine war. Effectively addressing these issues assures employees that management is prepared to align with their interests during challenging circumstances.

Liborius and Kiewitz (2022) underscore the importance of leaders cultivating the soft skill of humility. This attribute proves instrumental in proactively addressing the needs and desires of employees, reducing delays, and effectively addressing the escalating challenges associated with an increase in employee turnover intention within the firm. Engaging with the local community emerges as a noteworthy strategy, as proposed by Baba et al. (2021). Such engagement enables leaders to fulfil both personal and social responsibilities, fostering a sense of connectedness with the local community and promoting positive employee well-being in the workplace.

King et al. (2021) advocate for leaders’ continuous introspection at frequent intervals.

This reflective practice serves as a mechanism for leaders to scrutinize the potential negative impact of their adopted leadership practices on employees. Consequently, this approach facilitates the swift identification of corrective actions, ensuring a timely response to challenges posed by toxic leadership practices.

### 5.4 Discussion of the results

One of the major toxic leadership practices identified in the current study is the micromanagement of employees in every job position, as also affirmed in the study conducted by Franken & Plimmer (2019). Micromanagement refers to managers engaging in excessive control and involvement in the entire job process of employees. This leads to employees feeling burdened and being unable to focus on tasks. Furthermore, the independence of employees is reduced, significantly affecting employees’ perception of independence and autonomy in the workforce. The major strategies that have been suggested include managers engaging both in open communication and clearly establishing goals that need to be achieved by employees within a specified timeframe. This aspect is built on the concept of providing autonomy to employees and believing that employees will be able to complete the given job within a given timeframe covering aspects of both trust and accountability. A lack of adequate and reliable communication has also been considered a major challenge and was also highlighted in the study conducted by Yue et al. (2019). This can be done by ignoring sensitive information from employees, ignoring or taking into account employee feedback or having poor or irrelevant communication channels through which neither employees nor management are able to communicate from one stakeholder to another. Transparent and regular communication channels, which ensure that both management and employees know the relevant situation and what they expect in the foreseeable future, are also key here. Another major strategy that has been suggested is to focus not only on collecting employee feedback but also on taking corrective action based on such feedback, which ensures that employees feel their opinion is valued in the organization. This can also be ensured through managers actively listening to concerns. Unfair treatment has also been discussed here as one of the major attributes of toxic leadership practices and can include different aspects such as gender discrimination, favoritism, and the inadequate allocation of benefits to different stakeholders or the inadequate application of policies, with some employees receiving all benefits when compared to other groups who do not receive any benefit or do not obtain such benefit on time; these factors have also been extensively discussed in the study conducted by Lips-Wiersma et al. (2020). The strategy suggested here is to make regular and active attempts to reduce any instances of both discrimination and bias in the firm. Furthermore, the two aspects of diversity and inclusion can also be adopted here to improve the workforce dynamics of the organization and make each employee feel adequately represented in the firm. Additionally, equal compensation can be provided for different job roles, ensuring that equal promotion and career advancement opportunities are available for each candidate.

Bullying is also a major toxic leadership practice that was identified in the present study and includes both physical and domestic violence, humiliation with employees, and any form of mistreatment that is directed toward a particular gender or particular employee in the firm. This study takes into account each of these aspects collectively, which is a comprehensive picture of bullying in organizations covered by Ågotnes et al. (2021). A zero-tolerance policy has been suggested as a primary requirement that focuses on the mitigation of any such incident that might be reported throughout the organization. This policy must be further accompanied by the development and enactment of a proper reporting mechanism in which each employee is ensured that proper corrective action will be taken irrespective of the manager or other senior leader involved, which also builds the ethical foundation for a safe workplace in the organization. Another toxic leadership practice that has limited evidence in top management but rather has a significant presence in lower- and middle-level management is avoiding any responsibility for failure in the work process and shifting the responsibility for failure to subordinates, which has also been established in the study by Uygun & Gupta (2020). This aspect has been suggested to be addressed by introducing accountability among leaders, which will not only address this concern but also make leaders capable of addressing future challenges and building trust and loyalty among employees in the organization. This aspect also focuses on training both managers and employees to accept mistakes and consider mistakes as a learning opportunity that will aid them in addressing major challenges faced by the business. Collaboration has also been suggested as a major strategy here, but such collaboration needs to be performed to ensure that the manager is also accountable for the action.

A major challenge that has been noted as toxic leadership in organizations is the growth of authoritarian leadership qualities among managers. Authoritarian leadership discourages independent thinking and rewards the blind acceptance of orders from superiors; this is considered an extensive aspect, as noted in the study conducted by Pizzolitto et al. (2023). The above collaboration strategy suggested is considered a key strategy, it requires a shift in mindset in which employees are allowed to think independently, provide input, and develop creativity and critical thinking skills to address the challenges faced in an organization and hence engage in mutual growth. Additionally, the strategy of ensuring employee autonomy has also been suggested here to ensure a higher level of collaboration and address any challenge that might arise from managers attempting to control each aspect of the work process and employee job. Each of these challenges, from a broader aspect, highlights the main attribute of toxic leadership, which involves managers disregarding the well-being of employees as well as the emotions of employees and them being an indispensable part of the organization, as argued in the study by Deliu (2019). This aspect or the challenge of lack of empathy has been covered in the current study as a lack of consideration for employee well-being, lack of emotional intelligence, and undue focus on firm goals, with no consideration of employee emotions or challenges faced in the workplace. Training and development on emotional intelligence as well as the incorporation of a supportive work environment can aid in addressing such concerns and preparing both managers and employees to address future challenges without leaving the firm in time of exigency. Thus, a comprehensive analysis suggests that toxic leadership is a major barrier leading to employee turnover, but such can be addressed through systematic strategy deployment.

### 5.5 Implications for toxic leadership in the retail industry and lesson for the UAE retail industry

Toxic leadership in the retail industry encompasses a range of detrimental practices that adversely affect employee well-being and organizational dynamics. Characteristics such as authoritarianism, self-promotion, and indifference to employee concerns, as identified by Ali & Ullah (2023), contribute to a dearth of autonomy and participatory voice for employees. Mehmood et al. (2023b) emphasize the negative impact on employees’ psychological well-being and innovation, while Felicia et al. (2023) highlight traits leading to burnout and diminished job satisfaction. Aman et al. (2023) noted the direct influence of toxic leadership styles on turnover intentions, accentuated by a lack of promotion of positive organizational culture. Hossny et al. (2023) established how attributes such as workplace bullying and indifference undermine work quality, ultimately driving employees to leave. In the retail sector, Mossarah (2023) highlights issues such as inadequate compensation and flexibility, amplifying feelings of undervaluation and increasing turnover rates. Additionally, the pervasive impact of toxic leadership extends beyond turnover, affecting employee commitment, organizational performance, and overall job satisfaction, as emphasized by various studies, including those by Mehmood et al. (2023a), Choudhary & Saini (2021), and Kaymakcı et al. (2022). Overall, toxic leadership in retail poses a multifaceted challenge, influencing turnover rates, employee well-being, and organizational effectiveness.

In the realm of the retail sector in UAE, mitigating the perils of toxic leadership demands a comprehensive strategy. Ayça (2019) underscores the imperative of intensifying efforts to enhance organizational job satisfaction, advocating the adoption of authentic leadership to counter toxic attributes. Brender-Ilan and Sheaffer (2019) propose a nonradical shift by cultivating autonomy among subordinates, mitigating negative reactions. Eliyana and Ma’arif (2019) emphasize diverse transformational leadership styles that positively influence job satisfaction and organizational commitment. Hirschi and Spurk (2021) focus on employee advancement while acknowledging potential drawbacks such as migration. Ethical principles, as per Khizar et al. (2023), align with organizational objectives, complemented by Lee et al. (2023b), stressing leaders’ development of emotional intelligence. Social support, which is crucial during crises, is underscored by Lee et al. (2021). Mittal et al. (2022) advocated cultivating employee brand love and mitigating departures, and Olubiyi et al. (2019) stressed a friendly workplace and flexibility. Salau et al. (2018) propose transformational leadership for reassurance. Slatten et al. (2021) highlighted personal value alignment, Wei et al. (2023) suggested career advancement programs, and Coronado-Maldonado and Benítez-Márquez (2023) emphasized emotional competence. Liborius and Kiewitz (2022) stressed humility, Baba et al. (2021) advocated community engagement, and King et al. (2021) endorsed continuous leadership introspection. Integrating these strategies can create a resilient framework to mitigate toxic leadership impacts in the dynamic UAE retail landscape.

## 6. Conclusions

Toxic leadership involves the exhibition of different attributes that can negatively influence workforce dynamics in an organization by increasing the level of stress and workload while also engaging in gender-based discrimination and not making employees feel valued or part of the firm. The influence of toxic leadership has been noted not only on turnover intention among employees but also on organizational commitment, as shown by employees, and on feelings of job satisfaction. Hence, toxic leadership has been noted to influence both the physical and mental well-being of employees, all of which can reduce not only the willingness to perform in the organization but also the brand image as a reliable employer, which can negatively influence the survival rate of the brand. Several strategies have been noted by prior scholars, in which two major aspects are paying adequate attention to employee needs and aspirations while at the same time developing emotional intelligence and making employees feel valued as well as an integral part of the firm. This can be achieved through various strategies such as engaging in career advancement and training programs and promoting qualities of inclusive leadership. The adoption of an appropriate strategy depends on management and the balance between employee needs and the goals of the organization in the longer timeframe.

The results of the study, which show a positive correlation between toxic leadership and unfavourable employee outcomes, can assist organizations in realizing how urgently they must address detrimental leadership practices. Organizations can enhance employee well-being, productivity, and workplace culture by promoting healthy leadership practices, implementing targeted interventions, and comprehending these dynamics.

Retail managers and policymakers should put a high priority on creating positive leadership cultures by putting in place training courses that emphasize ethical behaviour, emotional intelligence, and effective communication. Early detection and treatment of toxic behaviors can be facilitated by anonymous feedback systems and routine evaluations. A respectful and safe workplace must be established, along with clear policies and support networks for impacted staff. It is recommended that policymakers implement industry-wide guidelines that promote best practices in leadership to foster organizational accountability. In addition to lowering employee attrition, funding leadership development and wellness programs will improve productivity, job satisfaction, and long-term organizational success in the fiercely competitive retail sector.

## 7. Limitations of the study

Limitations include certain technical and methodological aspects that can influence the validity and reliability of the results of the study. One of the major limitations of this study is the lack of inclusion of primary data, which has made it harder to empirically validate any findings established during the study. Additionally, another major limitation that has been noted is the methodological aspect itself. Journal articles that have not been published within the timeframe established and journal articles that are not open access may have had significant inferences that were excluded from the study to meet the inclusion and exclusion criteria. This systematic literature review’s methodological drawback is its dependence on certain databases, which might lead to publication bias by leaving out non-indexed or grey literature. In addition, another major limitation of this study is that it has not established a distinct focus on a specific industry or specific department, such as marketing and finance. Each of these sections might reveal a different leadership attribute, which might be termed toxic leadership by employees and hence may require distinct approaches, which was not considered in the present study. Thus, the general structure provides a comprehensive review but provides limited insight for specific departments within organizations and specific industries in the economic ecosystem.

## 8. Scope for future research

Future scholars should consider the current study as a major study that summarizes and synthesizes the work conducted by prior scholars regarding toxic leadership attributes, the impact of such attributes on turnover, and strategies that can be adopted to mitigate the challenges of toxic leadership in organizations. Furthermore, future scholars should attempt to conduct experimental studies in which employees who complain of toxic leadership are accounted for and the strategies suggested are applied to understand practical inferences of such strategies. Additionally, the empirical scope of the research should be expanded to include different industries and firms (both SMEs and large firms) to conduct a comparative analysis and analyse the strategies for each aspect. Moreover, future scholars should utilize this study as a reference point from which different attributes and strategies must be taken into consideration and utilized to construct more relevant and accurate survey and questionnaire instruments that can aid in furthering the related research. Future SLRS should focus on considering other major databases, as the current study only relied upon ScienceDirect. Finally, future scholars should consider each of the current study findings on a broader scale and attempt to apply them in different scenarios that are emerging in a business ecosystem, such as artificial intelligence integration and the growth of a hybrid work system.

## Ethical approval

This article does not contain any studies with human participants performed by any of the authors.

## Informed consent

This article does not contain any studies with human participants performed by any of the authors.

## Data Availability

The **PRISMA checklist**, **flowchart**, and **extended data (Table 1)** supporting this systematic review are publicly available in the Zenodo repository at: https://doi.org/10.5281/zenodo.17376520 Abhishek, S. (2025). **Repository name:** Zenodo **DOI:** https://doi.org/10.5281/zenodo.17376520 Abhishek, S. (2025). **License:** CC-BY 4.0 As this is a **Systematic Literature Review (SLR)**, no primary data were generated or analyzed. The extended materials (checklist, flowchart, and summary table) are provided for transparency and reproducibility.
