# An Examination of the Relationship between Perceived Toxic Leadership and Employee Turnover in the Retail Sector in the UAE

**DOI:** 10.12688/f1000research.171576.2

**Published:** 2026-06-23

**Authors:** Abhishek Shukla, BR Santosh, Sangeetha Vinod

**Affiliations:** 1 School of business, Manipal Academy of Higher Education - Dubai Campus, Academic City, Dubai, United Arab Emirates; 2 Manipal Institute of Commerce Finance and Economics, Manipal Academy of Higher Education, Manipal, Karnataka, India; 3 School of Business, Manipal Academy of Higher Education - Dubai Campus, Academic City, Dubai, United Arab Emirates

**Keywords:** Toxic Leadership, Job Satisfaction, Employee Turnover, Leadership

## Abstract

Toxic leadership or showcasing leadership skills, which are destructive to team spirit and individuals in the workplace, have been noted to significantly influence employee turnover. The purpose of the SLR (systematic literature review) is to investigate the impact of perceived toxic leadership on employee turnover, with the primary focus on the retail industry in the United Arab Emirates (UAE). Fifty journal articles were identified from a systematic analysis of the Science Direct database from 2018 to 2023 and have been included here to investigate toxic leadership, its influence on job satisfaction, organizational commitment, along with strategies that can be adopted to mitigate toxic leadership. This study finds that toxic leadership has a significant impact on turnover intention and organizational commitment of the employees. It affects both the mental and physical well-being of the employees, which leads to poor employee performance. Toxic leadership also negatively impacts the employer brand image and sustainability of the brand. The study outlines that to minimize the negative influence of toxic leadership, companies should provide sufficient consideration for the interests and goals of employees while also fostering emotional intelligence and ensuring that workers feel appreciated and an essential component of the company.

## 1. Introduction

### 1.1 Background for review

Toxic leadership, according to Padmanabhan (2021), is engaging in any leadership style that focuses on leaders not taking into consideration the interests and needs of employees but only on the completion of work objectives or engaging in meeting the interests of leaders or firms. Hence, this aspect involves any attempt to increase workload, job responsibilities, or job time without giving due focus on employees’ social and personal needs. Toxic leadership should be noted to emerge from any part of the organization’s hierarchy and needs to be taken into due consideration by management. This is critical as human resources are a crucial part of any organization. The negative influence of toxic leadership has been established in the study conducted by Felicia et al. (2023). The primary impact of toxic leadership is an increase in stress and a decrease in the physical and mental well-being of employees, which can lead to an increase in the level of burnout and ultimately to the decision to quit the firm to seek better opportunities or the same opportunities that do not cause the same level of stress. Hence, the impact of toxic leadership can be felt across different demographic aspects (age, gender, and work experience); thus, toxic leadership can be felt across all job positions in a firm.

An employee is considered to work more efficiently and continue working with the firm if he or she feels satisfied with the job role and firm, as highlighted in the study by Abd-Ellatif et al. (2021). Toxic leadership might lead to an increase in stress and workload, which negatively influences the level of employee satisfaction and hence makes employees unwilling to continue working. Employees may substitute satisfaction for better compensation, but such compensation is not expected to continue over a longer timeframe. Consequently, job satisfaction has become an inherent attribute that managers must focus upon to ensure employee well-being and target achievement. Another aspect is organizational commitment, which was evaluated by Choudhary & Saini (2021). Organization commitment refers to the psychological state of employees who are committed to the growth of the organization and focused on the achievement of job goals in a dedicated and timely manner. Commitment must be ensured by both management and employees. Management is noted to exhibit a lack of commitment owing to the adoption of toxic-leadership practices, which influence organizational commitment showcased by employees and hence influence their intention to stay with the firm for the foreseeable future. Toxic leadership is a major problem and needs to be addressed through dedicated strategies that, according to Lee et al. (2023a), shall primarily focus on management, focusing on the emotional needs and aspirations of employees and hence striking a balance between organizational goals and employee needs. This can be done either through the introduction of dedicated leadership skills such as transformational and inclusive skills or through the development of emotional intelligence among managers. Therefore, the major focus of each strategy is to ensure that managers can take into account employee needs as well and not cause any physical or mental damage to employees who are an inherent part of the organization.

Employees specifically need moral leadership to maintain a positive workplace culture, enhance psychological wellness, and maintain productivity. According to recent research, many organisations, societies, and nations suffer as a result of toxic leadership, a complex and harmful leadership style marked by unfavourable managerial practices. Further study on toxic leadership is still needed, even with the abundance of literature on the negative aspects of leadership and its effects on workers and organisations Akinyele and Chen (2024).

This research responds directly to the research agenda outlined above by Akinyele and Chen (2024), who suggested that further research in toxic leadership is needed in the context of different countries and work settings, as the authors highlighted that entire organisations, societies and even countries are affected by toxic leadership. By providing insights for leadership development and organisational policy reforms in a crucial and sometimes disregarded industry, this research advances our understanding of how toxic behaviours impact employee well-being, performance, and retention. The study offers novelty by synthesizing fragmented research on toxic leadership in the retail sector of the UAE, a context often overlooked. The SLR was developed primarily using the ScienceDirect database, which has an extensive repository of journal articles, to derive unique inferences from existing studies regarding different aspects of toxic leadership. ScienceDirect database was selected for this study as it provides broad access to excellent, peer-reviewed publications in the social sciences, psychology, and business management. It is the best resource for this comprehensive literature review. Its reliable search capabilities and reputation guarantee thorough coverage of pertinent research. However, one limitation of ScienceDirect is that it mostly contains articles from Elsevier, which may leave out pertinent research from other top databases and publishers.

This systematic review is inspired by the fact that the management of employees’ retention in the retail business and their leadership effectiveness is becoming relevant in the UAE, but empirical academic and/or journalistic evidence collected in this review was not necessarily retrieved in UAE based retail businesses. The final sample of articles was all studies carried out in various fields and countries, such as, health, hostel, manufacture, education, project environment, and retail environment. There were very few studies from the UAE or ‘Gulf’ setting.

Therefore, the aim of this review is not to make such direct causal conclusions for UAE retail organisations, but to combine the evidence from all around the world and to draw inferences from this about the implications in the UAE retail world. The UAE retail sector is especially suited to this context focusing, as the labour market environment in the sector is unique, including multiculturality, expatriation and high employee mobility, and an increase in service intensity. It should be understood, therefore, that the findings are conceptual transferable results that can be used to inform leadership practices and employee retention in UAE retail, not results from UAE specific data. This positioning adds to the external relevance of the review with the preservation of the methodological transparency related to the geographical distribution of the studies included.

### 1.2 Review objectives

• To examine the impact of perceived toxic leadership on employee turnover.
• To assess how employee satisfaction and organizational commitment are affected by toxic leadership.
• To investigate how shift timing and organizational structure influence the relationship between toxic leadership and employee turnover. To propose effective strategies to reduce the negative effects of toxic leadership types on employee turnover.

### 1.3 Research questions

• What is the impact of perceived toxic leadership on employee turnover?
• How are employee satisfaction and organizational commitment affected by toxic leadership? How does shift timing and organizational structure influence the relationship between toxic leadership and employee turnover? What are the different effective strategies for reducing the negative effects of toxic leadership types on employee turnover?

## 2. Inclusion and exclusion criteria

### 2.1 Criteria for inclusion

• All journal articles were published during the time period of 2018-2023.
• All the articles were free of full text.
• Each article is in accordance with the objectives of the study.

### 2.2 Criteria for exclusion

• All studies that were published before 2018 and were in different stages of publication were excluded.
• All studies that were not readily accessible or translatable to English were excluded.
• All abstracts, book chapters, conference papers, and entire books were excluded from the study.

## 3. Research methodology

### 3.1 Search strategy

This is a systematic literature review (SLR) that was performed by identifying, screening and synthesizing journal articles relevant to the study that were found primarily through the database ScienceDirect. Based on the characteristics above and with the consideration that there is a possibility of studying the relationship between toxic leadership and employee turnover ( Yasin et al., 2020; Mourão et al., 2020), it was decided to use ScienceDirect. Furthermore, the database provides built-in structure-based indexing schemas and rich search and filter options to facilitate transparency, consistency and replicability of the selection of the literature process.

ScienceDirect has excellent quality scholarly publications, but using only a single database can lead to publication and indexing bias as studies potentially relevant to the project not published there but in other indexing databases like Scopus, Web of Science, Emerald, SpringerLink, and Google-Scholar may be missed ( Yasin et al., 2020). In order to minimise this weakness, broad combination of various keywords, and a limited scope of inclusion and exclusion criteria were used to enhance the scope of retrieval from the chosen databases. Thus results should be considered within the framework of the evidence base included in the selection, but not in reference to all the literature. Future systematic literature reviews should explore the possibility of using multi-database search strategies, as this approach would give a more thorough search, minimize selection bias and would enhance methodological rigor ( Mourão et al., 2020).

### 3.2 Methods for study selection and appraisal

**Figure 1.**
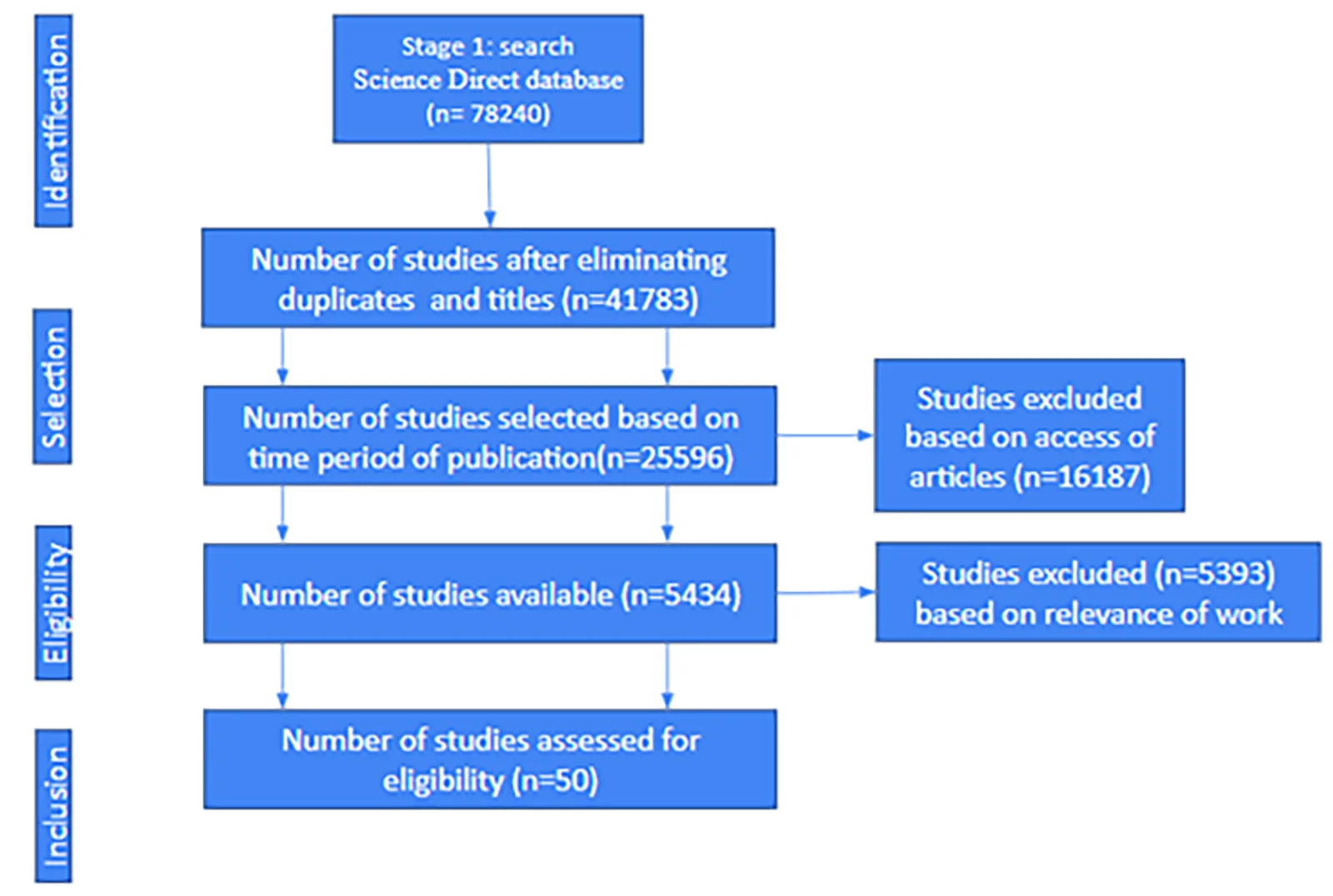
Methodology adopted in SLR: Different steps in performing the research.

The PRISMA research model established in the studies conducted by Page et al. (2021) and How et al. (2022) is the primary research methodology adopted in the study. An initial search of the ScienceDirect database using keyword strings established led to the initial selection of approximately 78240 journal articles. In the subsequent stage, all duplicates and titles were eliminated from the database that was developed in the initial study, which led to the inclusion of only 41783 articles in the database. Next, all the articles selected were reviewed based on the time period of publication (published from 2018 to 2023), which led to the identification of 25596 articles that met the inclusion criteria and were unique. The next step involved the exclusion of all such articles that were not open access (or not accessible by general readers who may not have access to paid journals or databases), which led to the further exclusion of 16187 articles and the selection of 5434 articles for the final database. The abstracts of the selected articles were manually reviewed to ensure that each was in accordance with the research objectives and could provide unique insights, which led to the further exclusion of 5384 articles and the selection of final 50 articles included in the review, which are listed in the table below. The PRISMA model below provides a graphical representation of the entire procedure. The process is illustrated in Figure 1.

From the above PRISMA model, we can observe that the initial search using the keywords provides 78240 results. Further out of these studies, 41783 studies were eliminated due to duplication of the research, hence, 25596 studies remained. From these studies, 16187 studies were removed due to a lack of access, resulting in 5434 studies remaining that were publicly accessible. Lastly, 5384 studies were eliminated due to lack of relevance to the present study, resulting in the selection of the final 50 articles.

In order to enhance transparency and methodological rigor, the studies selected were done according to PRISMA and a screening process was documented. However, as the strategy adopted was to use a single database, the methodological uniformity has been given more weight than the retrieval of all relevant studies, and there is the possibility of missing relevant studies.

## 4. Extraction and synthesis

The extracted data are summarized in **Table 1**. (Extended data)

Table 1 provides a brief summary of key data from the selected studies that includes author name(s), year, title, methodology and major observations. It facilitates comparison across studies and supports analysis by organizing relevant evidence systematically, aiding in the identification of patterns, trends, and gaps within the reviewed literature.

In addition to descriptive extraction, the thematic synthesis approach was used to find dominant themes, connections and patterns around the extracted themes across studies. Extracted findings were clustered based on similarity of concepts and then interpreted at macro level, related to broader categories about four different leadership behaviours and four different psychological outcomes as (1) leadership effectiveness, (2) employee’s psychological outcomes, (3) organisational commitment level, (4) turnover intention, and (5) mitigation mechanisms. This facilitated the review to transcend the sum it of its parts with regard to toxic leadership summarisation and to develop an integrated conceptual understanding of toxic leadership in organisations.

### 4.1 Geographical and sectoral distribution of included studies

The selected studies were also analysed by geographical region and industry activity to gain a clearer perspective on how these studies are applied and framed by their geographical and industry contexts. The evidence base was found to be spread across different countries as well as not restricted to UAE retail industry. The leadership outcomes selected for most of the studies were from the service sector, healthcare sector, hospitality sector and work organisations in general with few UAE based and retail specific studies included. This distribution is useful to note that all points derived in the present review take into account a wider pattern across the world of toxic leadership, and not just from the UAE. As a result, any recommendations for UAE retail would be context-dependent applications of the International evidence; and there is a need for further empirical studies to be undertaken within retail organisations directly in the UAE.

## 5. Review results and discussion

**Figure 2.**
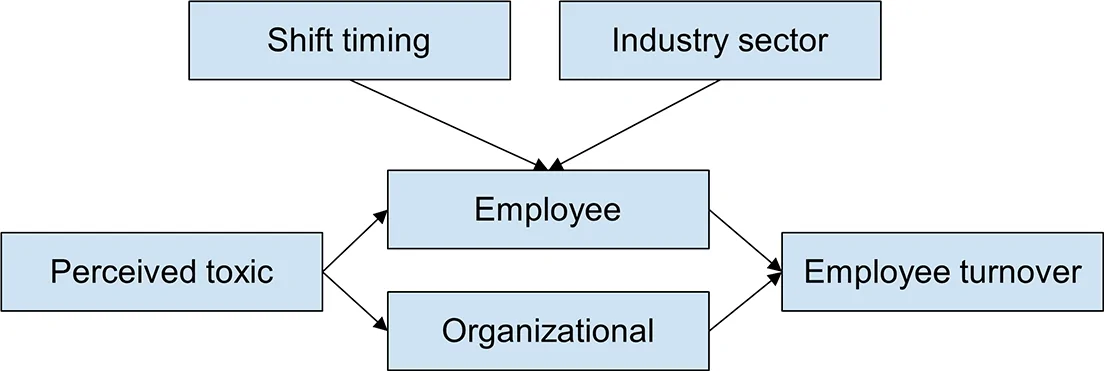
Conceptual framework.

The conceptual framework is presented in Figure 2.

### 5.1 Toxic leadership and employee turnover

In the contemporary corporate landscape, toxic leadership poses a formidable challenge for employees, as underscored by Ali and Ullah (2023). Emphasizing the importance of autonomy and participatory involvement in the workplace, employees find these essential aspects notably absent in toxic leadership environments, thereby fostering an environment conducive to increased employee turnover. Furthermore, Aman et al. (2023) assert that leadership styles significantly impact job-turnover intentions due to their influence on organizational culture. Toxic leadership, characterized by a lack of promotion of a positive organizational culture, contributes to elevated turnover rates across diverse job roles. Cortés-Denia et al. (2023) extend this perspective by highlighting the adverse effects of toxic leadership on employee engagement and commitment to achieving organizational objectives, ultimately propelling individuals to seek alternative employment in organizations that prioritize and respect social aspects of employee well-being.

The detrimental impact of toxic leadership extends beyond turnover rates, as noted by Felicia et al. (2023). Traits associated with toxic leadership contribute to employee burnout and diminish job satisfaction, thereby intensifying turnover intentions across various organizational levels. He et al. (2023) delve into the psychological ramifications of toxic leadership, emphasizing how it negatively influences the psychological attributes and behaviors of employees within the workplace. Considering the work of Holm et al. (2023), toxic leadership, manifested through workplace bullying and indifference to related reports, further impairs the quality of work, ultimately fostering an environment conducive to employee attrition over time.

Hossny et al. (2023) underscore the extensive role of toxic leadership, particularly its authoritarian attributes and self-promotional tendencies, in deteriorating organizational culture and diminishing employees’ willingness to remain in the retail industry. Lee et al. (2023b) contribute to this discourse by highlighting that a myopic focus on meeting job demands without addressing individual job aspects engenders a perception of undervaluation among employees, compelling them to seek alternative roles where their contributions are more recognized and appreciated.

Mehmood et al. (2023b) illuminate the detrimental impact of toxic leadership on employees’ psychological well-being and innovation within the workplace. The study establishes a direct negative correlation between toxic leadership attributes and the innovative output of affected employees. Moreover, toxic leadership qualities contribute to a decrease in employee creativity, while Mossarah (2023) underscores the adverse effects of such leadership on employee compensation and flexibility. The discrepancy between expected and offered salary and flexibility intensifies feelings of undervaluation among employees, fostering an environment conducive to organizational departure.

Obuobisa-Darko and Sokro (2023) identify leaders’ failure to align with employee interests and build mental resilience during challenging circumstances as indicative of toxic and insensitive leadership. This trait correlates with heightened turnover intentions among employees, particularly when confronted with the challenges posed by the pandemic. Padmanabhan (2021) accentuates the association between toxic leadership practices and increased stress levels among average employees, influencing their willingness to remain in the same position within the organization. Rafique et al. (2022) extend this perspective by linking toxic leadership practices, exacerbated by the challenges of the pandemic, to heightened job stress, especially in the context of adapting to hybrid work systems and integrating remote work into organizational processes.

Discussing the impact of toxic leadership on the employees, Salahat and Al-Hamdan (2022) identify a decrease in job satisfaction as a direct consequence of toxic leadership, establishing a correlation between reduced satisfaction and the intention to seek better opportunities elsewhere. Al-Jumaili et al. (2023) shed light on the pervasive issue of excessive workload as a common variable associated with toxic leadership. It becomes evident that toxic leadership practices encompass stress, reduced job satisfaction, and an increased workload, collectively impacting the workforce ( Goetz and Wald (2022); Jena et al. (2018)).

While considering the attributes of toxic leadership, Noor et al. (2023) identify gender discrimination and favouritism as prominent toxic leadership attributes that contribute to turnover among specific employee segments that perceive inadequate opportunities for advancement. On the other hand, Sokal et al. (2021) emphasize the lack of support from top management, whether in the absence of a developed strategy or failure to consider middle management proposals, as a toxic leadership practice. Discussing the personality traits of the leaders, Brender-Ilan and Sheaffer (2019) highlight the narcissistic traits exhibited by leaders as another facet of toxic leadership, emphasizing the imperative for prompt and effective management of such traits across all hierarchical positions within the organization.

### 5.2 Influence of employee satisfaction and organizational commitment on the impact of toxic leadership on employee turnover

Abd-Ellatif et al. (2021) assert that employee satisfaction has been adversely affected by heightened economic apprehensions since the COVID-19 pandemic. Within this context, toxic leadership exacerbates concerns among employees, amplifying fears about potential job impacts. Additionally, Adiguzel et al. (2020) posit a close correlation between the quality of leadership exhibited by managers and employee satisfaction. The failure of managers to instil motivation, coupled with detrimental behaviors, directly influences job satisfaction. Bezdrob and Šunje (2021) highlight the transient nature of job satisfaction, emphasizing the dynamic influence of leadership styles that necessitate ongoing monitoring.

Choudhary and Saini (2021) establish the pivotal role of organizational commitment in shaping job satisfaction. Recognizing the profound influence of organizational commitment on both employee turnover and job satisfaction due to toxic leadership underscores the necessity of considering organizational commitment when evaluating leadership impacts. Folakemi et al. (2018) extend this perspective by elucidating the influence of transformational and transactional leadership styles on both organizational commitment and job satisfaction, revealing universal applicability across permanent and temporary organizations and diverse industries.

Inegbedion et al. (2020) discern the differential impacts of toxic leadership attributes on job satisfaction, with certain attributes exerting a more pronounced influence, such as increased workloads and inadequate staffing levels, leading to diminished employee satisfaction. Kaymakcı et al. (2022) correlate the positive impacts of all variables on organizational performance, innovation, and a culture of innovation, emphasizing the necessity of mitigating toxic leadership attributes for organizational goal achievement. Kim et al. (2023) accentuate the universal nature of such influences across various pay levels and job positions, underscoring the pervasive impact experienced by all employees.

Labrague et al. (2018) contribute valuable insights into the universality of these influences across demographic variables, including gender, education, and work experience, reinforcing the generalizability of the relationship. Li et al. (2020) posit job satisfaction as a significant mediating variable in the nexus between various leadership styles and the intention to leave a job, further emphasizing its centrality in leadership impact assessments. Ly (2023) underscores the positive influence of inclusive leadership attributes on organizational commitment, suggesting the imperative for management to cultivate such attributes. Ryu et al. (2020) elucidate the emotional connection between employees and the organization, positing that negative satisfaction and commitment aspects significantly increase the likelihood of employee departure. In contrast, Van Rossenberg et al. (2022) challenge conventional notions of commitment as static, arguing for a dynamic understanding, while Jena et al. (2018) emphasize the interplay between employee satisfaction, commitment, and trust in management actions as key factors in reducing employee intentions to seek better opportunities elsewhere.

### 5.3 Toxic leadership mitigation strategies

Ayça (2019) advocates a substantive approach to ameliorate the ramifications of toxic leadership within the retail industry by intensifying efforts to enhance organizational job satisfaction. Embracing leadership styles, such as authentic leadership, proves instrumental in mitigating the adverse effects stemming from toxic leadership attributes. On the contrary, Brender-Ilan and Sheaffer (2019) propose a strategy in which, without necessitating a radical shift in leadership style, managers are encouraged to actively cultivate autonomy among subordinates in various job functions. This proactive measure serves to attenuate negative subordinate reactions resulting from toxic leadership.

Eliyana and Ma’arif (2019) endorse the adoption of diverse transformational leadership styles, citing their substantial impact on both job satisfaction and organizational commitment. This multifaceted influence, in turn, positively affects employee performance. Hirschi and Spurk (2021) posit a strategy focused on employee advancement and career progression, acknowledging potential drawbacks such as employee migration for superior opportunities. Simultaneously, prioritizing psychological well-being and demonstrating elevated trust in employees, as suggested by Jena et al. (2018), represent pivotal strategies for fostering employee engagement and a sense of organizational value. They also suggested that the HR department must avoid nepotism of any type while practising openness. Improving corporate culture and implementing forward-thinking HR procedures can promote pleasant working relationships between management and staff.

Khizar et al. (2023) underscore the significance of ethical principles and leadership adoption, emphasizing their critical role in guiding managerial actions. These principles align with organizational objectives while addressing the interests of various stakeholders, including employees. Complementing ethical considerations, Lee et al. (2023b) stress the importance of leaders developing emotional intelligence to comprehend employees’ social and psychological needs, collectively contributing to the mitigation of toxic leadership attributes. Lee et al. (2021) underscored social support, particularly in critical situations such as a civic pandemic, as essential for alleviating fear among employees and fostering heightened customer loyalty.

Mittal et al. (2022) highlighted the cultivation of employee brand love as a strategic imperative that positively impacts employee engagement and commitment while diminishing the propensity of employees to depart due to adverse leadership attributes. Creating a friendly workplace environment and affording flexibility, as advocated by Olubiyi et al. (2019), serves as a pivotal strategy applicable across all job positions, precluding perceptions of discrimination and favouritism. Salau et al. (2018) propose the adoption of transformational leadership attributes as a means to instil reassurance among employees impacted by toxic leadership, thereby redirecting their focus toward organizational goals. In order to create an environment of comfort, managers and supervisors must link the vision to a plan for achieving it and work with the staff to create an engaging and challenging vision.

Slatten et al. (2021) posit a significant strategic avenue wherein employees aligning their personal values with company values may establish a deeper connection with the organization. Such alignment potentially fosters increased engagement in management activities. This is because employees actively seek to diminish the adverse effects of prior toxic leadership practices. Furthermore, Wei et al. (2023) assert that the implementation of career advancement and training programs, including formal upskilling initiatives from dedicated certificate-providing institutions, is an effective measure for mitigating negative repercussions. Notably, these interventions directly benefit employees occupying lower and middle positions within the organizational hierarchy.

Coronado-Maldonado and Benítez-Márquez (2023) advocated the cultivation of emotional competence and emotional intelligence among leaders as a favourable strategy. This development equips leaders to navigate complex situations, ranging from the challenges posed by a pandemic to geopolitical conflicts such as the Ukraine war. Effectively addressing these issues assures employees that management is prepared to align with their interests during challenging circumstances.

Liborius and Kiewitz (2022) underscore the importance of leaders cultivating the soft skill of humility. This attribute proves instrumental in proactively addressing the needs and desires of employees, reducing delays, and effectively addressing the escalating challenges associated with an increase in employee turnover intention within the firm. Engaging with the local community emerges as a noteworthy strategy, as proposed by Baba et al. (2021). Such engagement enables leaders to fulfil both personal and social responsibilities, fostering a sense of connectedness with the local community and promoting positive employee well-being in the workplace.

King et al. (2021) advocate for leaders’ continuous introspection at frequent intervals.

This reflective practice serves as a mechanism for leaders to scrutinize the potential negative impact of their adopted leadership practices on employees. Consequently, this approach facilitates the swift identification of corrective actions, ensuring a timely response to challenges posed by toxic leadership practices.

### 5.4 Integrative conceptual analysis of toxic leadership and employee turnover

The findings reviewed in this study is not “an individual trait” of the leader that leads to turnover, but rather a multidimensional phenomenon of organisations that affects employee turnover in a manner where the psychological, behavioural, and organisational effects are interwoven. Throughout about all the the evidence, the pattern that arose was that the key characteristics of a leader’s toxic leadership behaviors – authoritarian leadership, hostility, too much monitoring, lack of emotional support, favouritism and employee participation – did not usually directly cause turnover intention. On the other hand, these leadership behaviours had a chain chain effect on the organisation which, in turn, gradually decreased employees’ desire to stay with it ( Ali & Ullah, 2023; Hossny et al., 2023; Noor et al., 2023; Pizzolitto et al., 2023; Brender-Ilan & Sheaffer, 2019).

The first major pathway found is the psychological deterioration pathway, through which one can increase stress, emotional exhaustion, burnout and decreased psychological well-being through toxic leadership. The outcomes decrease employee engagement and inspire withdrawal behaviours, which will finally result in push for departure ( Felicia et al., 2023; He et al., 2023; Padmanabhan, 2021; Rafique et al., 2022; Lee et al., 2023). The relational/commitment pathway, as identified in studies, is one where the toxic leadership negatively impacts the trust employees have in management leading to a decrease in organisational commitment and/or the relationship between the employee and the organizations’ goals ( Choudhary & Saini, 2021; Van Rossenberg et al., 2022; Ryu et al., 2020; Jena et al., 2018; Ly, 2023). The third pathway is the work environment pathway, which sees workplace trust and fairness decreased, as well as communication and autonomy in the work environment, due to toxic leadership ( Adiguzel et al., 2020; Abd-Ellatif et al., 2021; Inegbedion et al., 2020; Lips-Wiersma et al., 2020; Yue et al., 2019).

Importantly, of course, the review indicates that employee satisfaction and organisational commitment serve as mediators and not outcomes. Problematic leadership does not always lead to instant turnover; more often employees’ attitudes toward their workplace become increasingly dissatisfied, and his or her commitment to the organization begins to wane, which allows for a gradual escalation in intention to leave the organization. At the same time, the intensity of these outcomes seems moderated by the characteristics of the organisation such as leadership culture, organizational communication, being flexible and giving employees support ( Li et al., 2020; Choudhary & Saini, 2021; Kim et al., 2023; Goetz & Wald, 2022; Lee et al., 2021).

In addition, the synthesis concludes that the mitigation strategies that have appeared in the literature are conceptually grouped into 3 intervention fields, which are: (1) Development of leadership capacities (the concept of emotional intelligence, ethical leadership, humility), (2) Organisational practices oriented to employees (Participation, Recognition, Autonomy), (3) Support of the structure (Career progression, Feedback system, Inclusive policies). These results all support the view that toxic leadership can be considered as an organizational state that has been created through the interactions between the leadership behaviors, employees’ perceptions and the organizational culture ( Khizar et al., 2023; Lee et al., 2023; Liborius & Kiewitz, 2022; Hirschi & Spurk, 2021; Eliyana & Ma’arif, 2019; Slatten et al., 2021; Wei et al., 2023; King et al., 2020).

### 5.5 Discussion of the results

Thematic synthesis done in this review shows that the occurrence of individual instances of toxic behaviors must be analyzed as a process that affects employee outcomes, the working mechanism behind which is connected within the system of the organization. From the analyzed literature, three trends arose; failure of employee mental health, negative relationships between employees and employer, and loss of a supportive working environment. The above themes converge and demonstrate that toxic leadership is always associated with intentions to leave employees on various contexts of organisations. The conceptual integration also brings out patterns that explain how and why leadership behaviours result in employee retention outcome; not only summarizing previous findings.

One of the major toxic leadership practices identified in the current study is the micromanagement of employees in every job position, as also affirmed in the study conducted by Franken & Plimmer (2019). Micromanagement refers to managers engaging in excessive control and involvement in the entire job process of employees. This leads to employees feeling burdened and being unable to focus on tasks. Furthermore, the independence of employees is reduced, significantly affecting employees’ perception of independence and autonomy in the workforce. The major strategies that have been suggested include managers engaging both in open communication and clearly establishing goals that need to be achieved by employees within a specified timeframe. This aspect is built on the concept of providing autonomy to employees and believing that employees will be able to complete the given job within a given timeframe covering aspects of both trust and accountability. A lack of adequate and reliable communication has also been considered a major challenge and was also highlighted in the study conducted by Yue et al. (2019). This can be done by ignoring sensitive information from employees, ignoring or taking into account employee feedback or having poor or irrelevant communication channels through which neither employees nor management are able to communicate from one stakeholder to another. Transparent and regular communication channels, which ensure that both management and employees know the relevant situation and what they expect in the foreseeable future, are also key here. Another major strategy that has been suggested is to focus not only on collecting employee feedback but also on taking corrective action based on such feedback, which ensures that employees feel their opinion is valued in the organization. This can also be ensured through managers actively listening to concerns. Unfair treatment has also been discussed here as one of the major attributes of toxic leadership practices and can include different aspects such as gender discrimination, favoritism, and the inadequate allocation of benefits to different stakeholders or the inadequate application of policies, with some employees receiving all benefits when compared to other groups who do not receive any benefit or do not obtain such benefit on time; these factors have also been extensively discussed in the study conducted by Lips-Wiersma et al. (2020). The strategy suggested here is to make regular and active attempts to reduce any instances of both discrimination and bias in the firm. Furthermore, the two aspects of diversity and inclusion can also be adopted here to improve the workforce dynamics of the organization and make each employee feel adequately represented in the firm. Additionally, equal compensation can be provided for different job roles, ensuring that equal promotion and career advancement opportunities are available for each candidate.

Bullying is also a major toxic leadership practice that was identified in the present study and includes both physical and domestic violence, humiliation with employees, and any form of mistreatment that is directed toward a particular gender or particular employee in the firm. This study takes into account each of these aspects collectively, which is a comprehensive picture of bullying in organizations covered by Ågotnes et al. (2021). A zero-tolerance policy has been suggested as a primary requirement that focuses on the mitigation of any such incident that might be reported throughout the organization. This policy must be further accompanied by the development and enactment of a proper reporting mechanism in which each employee is ensured that proper corrective action will be taken irrespective of the manager or other senior leader involved, which also builds the ethical foundation for a safe workplace in the organization. Another toxic leadership practice that has limited evidence in top management but rather has a significant presence in lower- and middle-level management is avoiding any responsibility for failure in the work process and shifting the responsibility for failure to subordinates, which has also been established in the study by Uygun & Gupta (2020). This aspect has been suggested to be addressed by introducing accountability among leaders, which will not only address this concern but also make leaders capable of addressing future challenges and building trust and loyalty among employees in the organization. This aspect also focuses on training both managers and employees to accept mistakes and consider mistakes as a learning opportunity that will aid them in addressing major challenges faced by the business. Collaboration has also been suggested as a major strategy here, but such collaboration needs to be performed to ensure that the manager is also accountable for the action.

A major challenge that has been noted as toxic leadership in organizations is the growth of authoritarian leadership qualities among managers. Authoritarian leadership discourages independent thinking and rewards the blind acceptance of orders from superiors; this is considered an extensive aspect, as noted in the study conducted by Pizzolitto et al. (2023). The above collaboration strategy suggested is considered a key strategy, it requires a shift in mindset in which employees are allowed to think independently, provide input, and develop creativity and critical thinking skills to address the challenges faced in an organization and hence engage in mutual growth. Additionally, the strategy of ensuring employee autonomy has also been suggested here to ensure a higher level of collaboration and address any challenge that might arise from managers attempting to control each aspect of the work process and employee job. Each of these challenges, from a broader aspect, highlights the main attribute of toxic leadership, which involves managers disregarding the well-being of employees as well as the emotions of employees and them being an indispensable part of the organization, as argued in the study by Deliu (2019). This aspect or the challenge of lack of empathy has been covered in the current study as a lack of consideration for employee well-being, lack of emotional intelligence, and undue focus on firm goals, with no consideration of employee emotions or challenges faced in the workplace. Training and development on emotional intelligence as well as the incorporation of a supportive work environment can aid in addressing such concerns and preparing both managers and employees to address future challenges without leaving the firm in time of exigency. Thus, a comprehensive analysis suggests that toxic leadership is a major barrier leading to employee turnover, but such can be addressed through systematic strategy deployment.

### 5.6 Theoretical contribution for addressing toxic leadership and employee turnover

In addition to providing a synthesis of existing results, this systematic literature review makes contributions to the theory, by drawing an integrative conceptual interpretation that responds to the theoretical challenge of how toxic leadership can result in outcome of employee turnover. The paper proves that toxic leadership can not be viewed as a direct cause of turnover, but a multi-faceted condition in an organisation that acts as a mediating and moderating variable.

The presented integration of the reviewed studies led the authors to propose an integrative framework in which toxic leadership behaviours, such as authoritarian control, low emotional support, favouritism, workplace hostility, and decreased employee autonomy, create negative employee experiences which affect organisational outcomes ( Ali & Ullah, 2023; Hossny et al., 2023; Noor et al., 2023; Brender-Ilan & Sheaffer, 2019). These behaviours trigger a first-stage response that manifests in employees’ psychological responses to their work, such as stress, burnout, emotional exhaustion, and decreased wellbeing ( Felicia et al., 2023; Padmanabhan, 2021; He et al., 2023).

Later, the psychological impacts will affect relational outcomes through the lower job satisfaction, lower organisational commitment, which will further raise the intention of the employees to leave the organisation ( Abd-Ellatif et al., 2021; Choudhary & Saini, 2021; Li et al., 2020; Ryu et al., 2020). The framework builds on the power of these connections through employing the quality of communication, leadership culture, employee participation, flexibility and support systems in the organisation ( Lee et al., 2021; Jena et al., 2018; Slatten et al., 2021).

Lastly, intervention mechanisms related to emotional intelligence, ethical leadership, transformational leadership, employee empowerment, and career development can be a stopgap that—when it comes to turnover outcomes—might break this cycle ( Khizar et al., 2023; Lee et al., 2023; Eliyana & Ma’arif, 2019; Wei et al., 2023).

For this reason, the proposed framework builds on extant research by combining some of the disjointed results found in the literature into a cohesive explanatory structure in which employee turnover is seen as an indirect effect resulting from psychological, relational, and organizational processes, not as a direct consequence merely of the toxic leadership. This theory is expected to lay the groundwork for empirical studies in the broader organisational frameworks and within the UAE retail environment.

### 5.7 Implications for toxic leadership in the retail industry and lesson for the UAE retail industry

However, it is important to recognise that the evidence reviewed was not primarily from UAE retail organisations, before discussing implications for the retail sector in the UAE. Hence, the next discussion does not imply a direct generalisability, rather it is taken in an interpretive manner. The implementation and application of insights from foreign studies are treated sensibly when this is done in the specific UAE context due to the variations among national contexts in leadership behaviours, employee expectations, labour regulations and workforce type. However, it is possible to discern some recurring trends concerning toxic leadership, employee dissatisfaction, decreased organisational commitment and intentions to leave, which makes it interesting to explore the problem of leadership that leaders might encounter in UAE retail organisations.

Toxic leadership in the retail industry encompasses a range of detrimental practices that adversely affect employee well-being and organizational dynamics. Characteristics such as authoritarianism, self-promotion, and indifference to employee concerns, as identified by Ali & Ullah (2023), contribute to a dearth of autonomy and participatory voice for employees. Mehmood et al. (2023b) emphasize the negative impact on employees’ psychological well-being and innovation, while Felicia et al. (2023) highlight traits leading to burnout and diminished job satisfaction. Aman et al. (2023) noted the direct influence of toxic leadership styles on turnover intentions, accentuated by a lack of promotion of positive organizational culture. Hossny et al. (2023) established how attributes such as workplace bullying and indifference undermine work quality, ultimately driving employees to leave. In the retail sector, Mossarah (2023) highlights issues such as inadequate compensation and flexibility, amplifying feelings of undervaluation and increasing turnover rates. Additionally, the pervasive impact of toxic leadership extends beyond turnover, affecting employee commitment, organizational performance, and overall job satisfaction, as emphasized by various studies, including those by Mehmood et al. (2023a), Choudhary & Saini (2021), and Kaymakcı et al. (2022). Overall, toxic leadership in retail poses a multifaceted challenge, influencing turnover rates, employee well-being, and organizational effectiveness.

In the realm of the retail sector in UAE, mitigating the perils of toxic leadership demands a comprehensive strategy. Ayça (2019) underscores the imperative of intensifying efforts to enhance organizational job satisfaction, advocating the adoption of authentic leadership to counter toxic attributes. Brender-Ilan and Sheaffer (2019) propose a nonradical shift by cultivating autonomy among subordinates, mitigating negative reactions. Eliyana and Ma’arif (2019) emphasize diverse transformational leadership styles that positively influence job satisfaction and organizational commitment. Hirschi and Spurk (2021) focus on employee advancement while acknowledging potential drawbacks such as migration. Ethical principles, as per Khizar et al. (2023), align with organizational objectives, complemented by Lee et al. (2023b), stressing leaders’ development of emotional intelligence. Social support, which is crucial during crises, is underscored by Lee et al. (2021). Mittal et al. (2022) advocated cultivating employee brand love and mitigating departures, and Olubiyi et al. (2019) stressed a friendly workplace and flexibility. Salau et al. (2018) propose transformational leadership for reassurance. Slatten et al. (2021) highlighted personal value alignment, Wei et al. (2023) suggested career advancement programs, and Coronado-Maldonado and Benítez-Márquez (2023) emphasized emotional competence. Liborius and Kiewitz (2022) stressed humility, Baba et al. (2021) advocated community engagement, and King et al. (2021) endorsed continuous leadership introspection. Integrating these strategies can create a resilient framework to mitigate toxic leadership impacts in the dynamic UAE retail landscape.

## 6. Conclusions

This review supplements previous summary descriptions of toxic leadership by factorial systematizing the evidence in a conceptual model surrounding the indirect nature of the relationship between leadership practices and employee turnover. The theoretical positioning reinforces the understanding of the employees outcomes as intertwined organisational processes to give grounds for the future empirical testing.

Toxic leadership involves the exhibition of different attributes that can negatively influence workforce dynamics in an organization by increasing the level of stress and workload while also engaging in gender-based discrimination and not making employees feel valued or part of the firm. The influence of toxic leadership has been noted not only on turnover intention among employees but also on organizational commitment, as shown by employees, and on feelings of job satisfaction. Hence, toxic leadership has been noted to influence both the physical and mental well-being of employees, all of which can reduce not only the willingness to perform in the organization but also the brand image as a reliable employer, which can negatively influence the survival rate of the brand. Several strategies have been noted by prior scholars, in which two major aspects are paying adequate attention to employee needs and aspirations while at the same time developing emotional intelligence and making employees feel valued as well as an integral part of the firm. This can be achieved through various strategies such as engaging in career advancement and training programs and promoting qualities of inclusive leadership. The adoption of an appropriate strategy depends on management and the balance between employee needs and the goals of the organization in the longer timeframe.

The results of the study, which show a positive correlation between toxic leadership and unfavourable employee outcomes, can assist organizations in realizing how urgently they must address detrimental leadership practices. Organizations can enhance employee well-being, productivity, and workplace culture by promoting healthy leadership practices, implementing targeted interventions, and comprehending these dynamics.

The theoretical contributions of this review are in the form of a proposed ‘integrated’ interpretation, whereby toxic leadership affects employee turnover indirectly via various assumed mechanisms of employee satisfaction, organisational commitment and workplace experience. The conceptual approach is a novel approach that adds to the scholarship on leadership in that employee outcomes are seen as a series of interconnected organisational responses, instead of as standalone outcomes.

Retail managers and policymakers should put a high priority on creating positive leadership cultures by putting in place training courses that emphasize ethical behaviour, emotional intelligence, and effective communication. Early detection and treatment of toxic behaviors can be facilitated by anonymous feedback systems and routine evaluations. A respectful and safe workplace must be established, along with clear policies and support networks for impacted staff. It is recommended that policymakers implement industry-wide guidelines that promote best practices in leadership to foster organizational accountability. In addition to lowering employee attrition, funding leadership development and wellness programs will improve productivity, job satisfaction, and long-term organizational success in the fiercely competitive retail sector.

## 7. Limitations of the study

Limitations include certain technical and methodological aspects that can influence the validity and reliability of the results of the study. One of the major limitations of this study is the lack of inclusion of primary data, which has made it harder to empirically validate any findings established during the study. Additionally, another major limitation that has been noted is the methodological aspect itself. Journal articles that have not been published within the timeframe established and journal articles that are not open access may have had significant inferences that were excluded from the study to meet the inclusion and exclusion criteria. This systematic literature review’s methodological drawback is its dependence on certain databases, which might lead to publication bias by leaving out non-indexed or grey literature. Moreover, exclusive access to ScienceDirect might have led to a publication bias and reduced literature coverage as the studies not published in the above-mentioned platform are not available. Despite the choice of the database for access to high quality, peer-reviewed studies, there may be an overlook of other indexing platforms, which could then limit the penitentiary synthesis. Future reviews should be more robust, reproducible and provide more context by using multiple databases and additional search strategies. In addition, another major limitation of this study is that it has not established a distinct focus on a specific industry or specific department, such as marketing and finance. Each of these sections might reveal a different leadership attribute, which might be termed toxic leadership by employees and hence may require distinct approaches, which was not considered in the present study.

This is one more drawback: the lack of context representation. The review focuses on the UAE retail industry, but the studies that have been included are primarily gathered from international and across-industry contexts. Consequently, the conclusions in the findings should not be seen as direct empirical evidence of toxic leadership qualities that exist in UAE retail organizations. It would be beneficial for future studies to implement a more specific database strategy and add search terms specific to the respective geographic areas when building up the retail evidence base in order to further narrow the scope for UAE specific studies. Thus, the general structure provides a comprehensive review but provides limited insight for specific departments within organizations and specific industries in the economic ecosystem.

## 8. Scope for future research

Future scholars should consider the current study as a major study that summarizes and synthesizes the work conducted by prior scholars regarding toxic leadership attributes, the impact of such attributes on turnover, and strategies that can be adopted to mitigate the challenges of toxic leadership in organizations. Furthermore, future scholars should attempt to conduct experimental studies in which employees who complain of toxic leadership are accounted for and the strategies suggested are applied to understand practical inferences of such strategies. Additionally, the empirical scope of the research should be expanded to include different industries and firms (both SMEs and large firms) to conduct a comparative analysis and analyse the strategies for each aspect. Moreover, future scholars should utilize this study as a reference point from which different attributes and strategies must be taken into consideration and utilized to construct more relevant and accurate survey and questionnaire instruments that can aid in furthering the related research. Future SLRS should focus on considering other major databases, as the current study only relied upon ScienceDirect. Finally, future scholars should consider each of the current study findings on a broader scale and attempt to apply them in different scenarios that are emerging in a business ecosystem, such as artificial intelligence integration and the growth of a hybrid work system.

## Ethical approval

This article does not contain any studies with human participants performed by any of the authors.

## Informed consent

This article does not contain any studies with human participants performed by any of the authors.

## Data Availability

The **PRISMA checklist**, **flowchart**, and **extended data (Table 1)** supporting this systematic review are publicly available in the Zenodo repository at: https://doi.org/10.5281/zenodo.17376520 [ Abhishek, S. (2025)]. **Repository name:** Zenodo **DOI:** https://doi.org/10.5281/zenodo.17376520 [ Abhishek, S. (2025)]. **License:** CC-BY 4.0 As this is a **Systematic Literature Review (SLR)**, no primary data were generated or analyzed. The extended materials (checklist, flowchart, and summary table) are provided for transparency and reproducibility.
