# Supplementary material for: An Examination of the Relationship between Perceived Toxic Leadership and Employee Turnover in the Retail Sector in the UAE
Source: F1000Res. 2026 Jun 23;14:1398. Originally published 2025 Dec 15. [Version 2] doi: 10.12688/f1000research.171576.2 (PMC13550894; doi:10.12688/f1000research.171576.2)
Supplement: Peer review history

# Reviewer report: Reviewer response for version 2

**DOI:** 10.5256/f1000research.203135.r496042

**Published:** 2026-07-07

**Authors:** Aarti Sharma

**Affiliations:** 1 IIHMR UNIVERSITY, Jaipur, Rajasthan, India

No further comments

Are the rationale for, and objectives of, the Systematic Review clearly stated?

Yes

Is the statistical analysis and its interpretation appropriate?

Yes

If this is a Living Systematic Review, is the ‘living’ method appropriate and is the search schedule clearly defined and justified? (‘Living Systematic Review’ or a variation of this term should be included in the title.)

Not applicable

Are sufficient details of the methods and analysis provided to allow replication by others?

Yes

Are the conclusions drawn adequately supported by the results presented in the review?

Yes

Reviewer Expertise:

Work Engagement, Well- being, Retention, Recruitment, Sustainable Development, Knowledge Management.

I confirm that I have read this submission and believe that I have an appropriate level of expertise to confirm that it is of an acceptable scientific standard.

# Reviewer report: Reviewer response for version 2

**DOI:** 10.5256/f1000research.203135.r496041

**Published:** 2026-07-06

**Authors:** Yustisia Kristiana

**Affiliations:** 1 Universitas Pelita Harapan, Tangerang, Indonesia

I confirm that I have read this submission and believe that I have an appropriate level of expertise to confirm that it is of an acceptable scientific standard.

 The results are clearly and accurately presented, and the conclusions are logical and fully supported by the data.

Are the rationale for, and objectives of, the Systematic Review clearly stated?

Yes

Is the statistical analysis and its interpretation appropriate?

Not applicable

If this is a Living Systematic Review, is the ‘living’ method appropriate and is the search schedule clearly defined and justified? (‘Living Systematic Review’ or a variation of this term should be included in the title.)

Not applicable

Are sufficient details of the methods and analysis provided to allow replication by others?

Partly

Are the conclusions drawn adequately supported by the results presented in the review?

Partly

Reviewer Expertise:

NA

I confirm that I have read this submission and believe that I have an appropriate level of expertise to confirm that it is of an acceptable scientific standard.

# Reviewer report: Reviewer response for version 1

**DOI:** 10.5256/f1000research.189197.r479396

**Published:** 2026-05-21

**Authors:** Aarti Sharma

**Affiliations:** 1 IIHMR UNIVERSITY, Jaipur, Rajasthan, India

The manuscript provides a well-organized systematic literature review on the relationship between perceived toxic leadership and employee turnover in the UAE retail sector. It addresses a relevant organizational issue with significant academic and practical implications. The research objectives are clearly defined and aligned with the review's focus.

 Employing the PRISMA framework enhances the study's methodological rigor, with systematic descriptions of article selection and screening. The review efficiently synthesizes literature, offering insights into how toxic leadership affects employee turnover intention, organizational commitment, employee well-being, and sustainability. The findings are logically presented, supported by evidence, and lead to coherent conclusions.

 Moreover, the manuscript highlights practical implications for organizations in areas such as leadership practices, employee engagement, emotional intelligence, and workplace culture. The writing is clear and accessible. Overall, the study significantly contributes to existing knowledge and is suitable for indexing in its current form.

Are the rationale for, and objectives of, the Systematic Review clearly stated?

Yes

Is the statistical analysis and its interpretation appropriate?

Yes

If this is a Living Systematic Review, is the ‘living’ method appropriate and is the search schedule clearly defined and justified? (‘Living Systematic Review’ or a variation of this term should be included in the title.)

Not applicable

Are sufficient details of the methods and analysis provided to allow replication by others?

Yes

Are the conclusions drawn adequately supported by the results presented in the review?

Yes

Reviewer Expertise:

Work Engagement, Well- being, Retention, Recruitment, Sustainable Development, Knowledge Management.

I confirm that I have read this submission and believe that I have an appropriate level of expertise to confirm that it is of an acceptable scientific standard.

# Reviewer report: Reviewer response for version 1

**DOI:** 10.5256/f1000research.189197.r462237

**Published:** 2026-03-06

**Authors:** Yustisia Kristiana

**Affiliations:** 1 Universitas Pelita Harapan, Tangerang, Indonesia

This article is a Systematic Literature Review (SLR) that aims to examine the relationship between perceived toxic leadership and employee turnover, focusing on the retail sector in the United Arab Emirates (UAE). Using the PRISMA approach and the ScienceDirect database for publications from 2018 to 2023, the author selected 50 articles for systematic analysis. The review shows that toxic leadership negatively affects important organizational factors, such as job satisfaction, organizational commitment, and psychological well-being, and also contributes to higher turnover intention. The article also identifies several mitigation strategies that organizations can apply, including strengthening transformational leadership, developing emotional intelligence, promoting open communication, and implementing anti-bullying policies.

 However, there are several key issues that need attention:

**Introduction:** The contextual focus on the UAE retail sector is not clearly shown. Most of the reviewed studies come from different countries and industries, so there is little specific analysis reflecting the unique characteristics of UAE retail. The author should clarify how many studies are actually UAE-based and consider a sub-analysis or adjust the research framing to match the available evidence.

**Methodology:** Using only the ScienceDirect database may cause publication bias and limit the coverage of literature. Stronger methodological justification and explicit acknowledgment of limitations are needed. There are inconsistencies in the number of excluded articles between the text and the diagram, which should be corrected for accurate, one part says 5,384 were excluded, while another says 5,393.

**Results and Discussion:** The synthesis of findings is still mostly descriptive, summarizing studies one by one without showing thematic integration or deeper conceptual patterns. The author should add a more integrative conceptual synthesis.

**Theoretical Contribution:** The article needs stronger theoretical contribution so that it is not just a literature summary but also provides conceptual value, for example by developing an integrative framework or a conceptual model based on the synthesis.

Are the rationale for, and objectives of, the Systematic Review clearly stated?

Yes

Is the statistical analysis and its interpretation appropriate?

Not applicable

If this is a Living Systematic Review, is the ‘living’ method appropriate and is the search schedule clearly defined and justified? (‘Living Systematic Review’ or a variation of this term should be included in the title.)

Not applicable

Are sufficient details of the methods and analysis provided to allow replication by others?

Partly

Are the conclusions drawn adequately supported by the results presented in the review?

Partly

Reviewer Expertise:

The results are clearly and accurately presented, and the conclusions are logical and fully supported by the data.

I confirm that I have read this submission and believe that I have an appropriate level of expertise to confirm that it is of an acceptable scientific standard, however I have significant reservations, as outlined above.

# Response

**Published:** 2026-06-03

**Authors:** Abhishek Shukla

**Affiliations:** School of Business, Manipal Academy of Higher Education - Dubai Campus, Academic City, Dubai, United Arab Emirates

We sincerely thank the reviewer for the valuable comments and constructive suggestions. The manuscript has been carefully revised, and all changes have been highlighted in the attached tracked-changes version.

**Comment 1: Introduction**

**Reviewer Comment:**

 The contextual focus on the UAE retail sector is not clearly shown. Most of the reviewed studies come from different countries and industries, so there is little specific analysis reflecting the unique characteristics of UAE retail.

**Response:**

 Thank you for this observation. The introduction has been revised to better frame the study within the UAE retail context. In order to clarify the relevance of the review to the UAE retail sector, a discussion has been added prior to the objectives of the review. This acknowledges the limited availability of UAE-specific studies. As part of the improvement of transparency regarding the evidence base, a subsection has been added describing the geographical and sectoral distribution of the included studies.

****

**Comment 2: Methodology**

**Reviewer Comment**:

 Using only the ScienceDirect database may cause publication bias and limit the coverage of literature. Stronger methodological justification and explicit acknowledgment of limitations are needed. There are inconsistencies in the number of excluded articles between the text and the diagram.

**Response:**

 Thank you for sharing this valuable suggestion. The methodology justification for choosing ScienceDirect has been strengthened in Section 3.1 (Search Strategy). Also, Section 3.2 has been revised, and a new paragraph acknowledges the limitations associated with the use of a single database, including potential publication bias and limited literature coverage. As part of the revision process, the screening and exclusion figures have also been reviewed and corrected throughout the manuscript to ensure consistency.

**Comment 3: Results and Discussion**

**Reviewer Comment:**

 The synthesis of findings is still mostly descriptive, summarizing studies one by one without showing thematic integration or deeper conceptual patterns.

**Response:**

 We appreciate the recommendation. An integrated thematic analysis of the reviewed studies has been added to the Extraction and Synthesis section. A new subsection on the geographical and sectoral distribution of included studies has been added, in addition to expanding Sections 5.4 and 5.5 to provide a deeper conceptual discussion of the relationships between toxic leadership, employee outcomes, organizational commitment, and turnover intention.

****

**Comment 4: Theoretical Contribution**

**Reviewer Comment:**

 The article needs stronger theoretical contribution so that it is not just a literature summary but also provides conceptual value.

**Response:**

 Thank you for your constructive suggestion. A dedicated section 5.6 has been added to strengthen the theoretical contribution titled "Theoretical Contribution to Addressing Toxic Leadership and Employee Turnover". Based on the literature review, this section presents an integrative conceptual framework. In addition, sections 5.7, 6, and 7 have also been revised to align the implications, conclusions, and future research directions with the proposed framework.

 Thank you for providing us with such thoughtful and constructive feedback. These revisions have significantly improved the manuscript's methodological rigor, theoretical contribution, contextual clarity, and overall scholarly significance.
